# From political pledges to quantitative mapping of climate mitigation plans: Comparison of two European cities

**DOI:** 10.1186/s13021-023-00236-y

**Published:** 2023-09-06

**Authors:** Ivonne Albarus, Giorgia Fleischmann, Patrick Aigner, Philippe Ciais, Hugo Denier van der Gon, Rianne Droge, Jinghui Lian, Miguel Andrey Narvaez Rincon, Hervé Utard, Thomas Lauvaux

**Affiliations:** 1 Laboratoire des Sciences du Climat et de l’Environnement (LSCE), IPSL, CEA-CNRS-UVSQ, Université Paris-Saclay, 91191 Gif sur Yvette Cedex, France; 2 Origins.earth, Suez Group, 92040 Paris La Défense, France; 3 Economics and Political Science, SciencesPo, Paris, France; 4 Environmental Sensing and Modeling, Technical University of Munich (TUM), Munich, Germany; 5 Department of Climate, Air and Sustainability, TNO, Utrecht, The Netherlands; 6 GSMA, UMR 7331, University of Reims Champagne Ardenne, Reims, France

**Keywords:** Climate action plan, Greenhouse gases, Fossil fuel emissions, Urban planning, Climate neutrality, Emission inventory

## Abstract

****Background**:**

Urban agglomerates play a crucial role in reaching global climate objectives. Many cities have committed to reducing their greenhouse gas emissions, but current emission trends remain unverifiable. Atmospheric monitoring of greenhouse gases offers an independent and transparent strategy to measure urban emissions. However, careful design of the monitoring network is crucial to be able to monitor the most important sectors as well as adjust to rapidly changing urban landscapes.

****Results**:**

Our study of Paris and Munich demonstrates how climate action plans, carbon emission inventories, and urban development plans can help design optimal atmospheric monitoring networks. We show that these two European cities display widely different trajectories in space and time, reflecting different emission reduction strategies and constraints due to administrative boundaries. The projected carbon emissions rely on future actions, hence uncertain, and we demonstrate how emission reductions vary significantly at the sub-city level.

****Conclusions**:**

We conclude that quantified individual cities’ climate actions are essential to construct more robust emissions trajectories at the city scale. Also, harmonization and compatibility of plans from various cities are necessary to make inter-comparisons of city climate targets possible. Furthermore, dense atmospheric networks extending beyond the city limits are needed to track emission trends over the coming decades.

**Supplementary Information:**

The online version contains supplementary material available at 10.1186/s13021-023-00236-y.

## Background

Urban settlements represent a large fraction of the world’s population. Currently, cities with more than 300.000 inhabitants are host to 59% of the global population, and their share is projected to increase to 68% over the next three decades [1]. Urban areas account for about two-thirds of global primary energy use and almost half of the energy-related direct $\textrm{CO}_2$ emissions [2]. These emissions are projected to rise in the decades to come [3]. In their latest report, the IPCC [4] confirms the impact of cities on global emissions and stresses their significance for swift and aggressive measures to support emission mitigation objectives at the national level. Local governments are, therefore, fundamental players in climate mitigation programs, impacting the carbon footprint of millions [5]. Thus, cities can play a critical role in implementing mitigation actions and achieving the Sustainable Development Goals (SDGs) [6]. Indeed, large and medium-sized metropolitan areas have started coordinating their efforts within international alliances. More than 10.000 cities have already joined the Global Covenant of Mayors (GCoM) alliance [7]. Many more signed up for multiple other climate alliances or initiatives (e.g., ICLEI [8], C40 [9], etc.) with the goal of sharing tools and experiences, and maximizing impact for climate action. Those alliances cover a large share of global emissions, with less than 100 city members of the C40 Cities initiative representing 10% of global emissions [5]. Thanks to those initiatives, cities now publish their emission inventories and climate mitigation plans, for instance, through the Carbon Disclosure Project [10], making cities accountable for their targets globally and locally [11]. A recent study has shown that participation in transnational climate governance is associated with a 1,6% reduction in annual emissions and that 84% of those participating cities have reduced their emissions between 2001 and 2018, while only 35% of non-participating cities have achieved a net reduction [12].

Climate neutrality has become a common goal, and many European cities try to follow the EU’s climate targets and strive for net-zero emissions between 2030 and 2050 [5, 13]. Efforts are typically concentrated on the deployment of renewables, as actions within the energy sector provide the highest reduction potential to save considerable amounts of $\textrm{CO}_2$ emissions. REN21 and its participants have found that globally 617 cities included a 100% renewable energy target in their objectives [14]. The second most frequent focus is on transport, for which the aim is to incentive the shift towards ’soft mobility’ through the development of cycle lanes and the use of electric vehicles, and last but not least, the increase of public awareness [5, 15, 16].

However, the quantification of mitigation policies is very often incomplete or unavailable, raising serious concerns about how and if climate mitigation targets will be achieved. In the Fifth Assessment Report (AR5) of the Intergovernmental Panel on Climate Change [3], the limited knowledge about emission reduction potentials from urban climate policies and strategies raised serious concerns about their achievability, questioning the integrity of climate targets. Milojevic-Dupont and Creutzig [17] additionally point out the critical disagreement about the resulting effects of mitigation actions. Current emissions inventory methods do not include the evaluation of policies that aim to reduce emissions [18]. This uncertainty around climate mitigation policies is contradictory since the latter should be precisely based on their mitigation potential [19].

In order to design robust climate policies, more detailed and timely information on emission trends is needed to support decision-makers [20]. Previous studies have already largely underlined the shortcomings of the cities’ Self-Reported Inventories (SRIs) due to the disparate and inconsistent nature of self-reported data [21], missing spatial information, or inconsistencies across carbon accounting protocols [22]. These studies have shown that SRIs alone are not sufficiently accurate for monitoring urban emissions. However, atmospheric observations are a valuable addition to monitoring Greenhouse Gas (GHG) emissions over time. With a strategy similar to air quality monitoring, atmospheric carbon monitoring has been proven to be very effective in providing timely and robust emission trends [23–26]. This hybrid approach integrates information from both city inventories and atmospheric observations, such as those presented by Mueller and colleagues [24], offering a calibrated and verified solution to validate the current emissions trends at the city scale. One further technique, known as atmospheric inversion, aims to reduce the current delays in annual inventories (typically 3–4 years behind real-time), provide spatial information on emissions, foster standardized methodologies in reporting, and provide scalable uniform and accurate information [24]. Based on atmospheric measurements collected in and around the metropolitan areas, this technology continuously infers direct emissions of the main greenhouse gases through the comparison of simulations from atmospheric transport models to concentrations from atmospheric GHG measurements [27–29]. Atmospheric methods are being implemented over several European cities (Paris, Munich, Zurich,...), offering a similar level of transparency concerning methods and input data but a higher level of evaluation thanks to calibrated atmospheric data [30]. Administrative boundaries delineate a fraction of the city emissions (e.g., Los Angeles, Paris) or cover a much larger area extending beyond the urban land. However, atmospheric inversions are able to constrain emission budgets within atmospheric measurement networks, not necessarily matching the political boundaries used for the inventories. For an atmospheric monitoring system, disentangling Paris’ emissions from its neighboring cities will, therefore, require high-resolution mapping capabilities [25]. Nevertheless, these systems have shown that current capabilities allow local governments to monitor changes over time [23, 31] or even quantify the short-term impacts of mobility restrictions [25, 29].

This study aims to shed new light on monitoring future emissions through an innovative approach that leverages existing emissions products and incorporates information from the city’s climate plan. While existing gridded emissions products offer valuable insights into past emissions, they do not account for future emission changes. To bridge this gap, we present projected emission maps for the target years 2030 and 2050. Our approach comprehensively considers the political and technical aspects of climate action plans, providing essential information for planning future monitoring systems. We assert that robust mapping of present and future emissions is essential for effectively tracing reduction trends at both the sectoral and spatial level. Moreover, this comprehensive mapping will serve as a foundational resource for designing sustainable and enduring city observation networks. By offering insights into projected emissions, our study aims to contribute to the development of informed and targeted climate policies and enables cities to take effective mitigation actions.

**Table 1:** Statistical comparison of Paris, France, and Munich, Germany

	Paris	Munich
Inhabitants (2019)	2.2 M [32]	1.5 M [33]
Surface	105 $km^2$ [32]	310 $km^2$ [34]
Density	20.952 inhabitants/$km^2$	4.839 inhabitants/$km^2$
GDP (2018) [35]	685,67 B€/year	189,16 B€/year
Mean annual temperature from 1991–2021	11,7$^{\circ }$C [36]	8.8$^{\circ }$C [37]
Seasonal amplitude between 1991–2021	15,5$^{\circ }$C [36]	18,8$^{\circ }$C [37]

We selected two European cities with distinct characteristics in terms of size, geographic location, and climate strategies. The goal of this study is to provide information on emissions trends up to 2030 and 2050 to project emissions changes with sectoral and spatial granularity. We base our projected maps on individual and sectoral climate actions outlined in the climate action plans. This type of projection provides essential information for the design of long-lasting urban atmospheric monitoring networks aiming to provide independent information on emission trends. Continuous monitoring will help quantify the impacts of mitigation actions, strengthen climate policies, and increase their effectiveness at the city level.

**Fig. 1:**
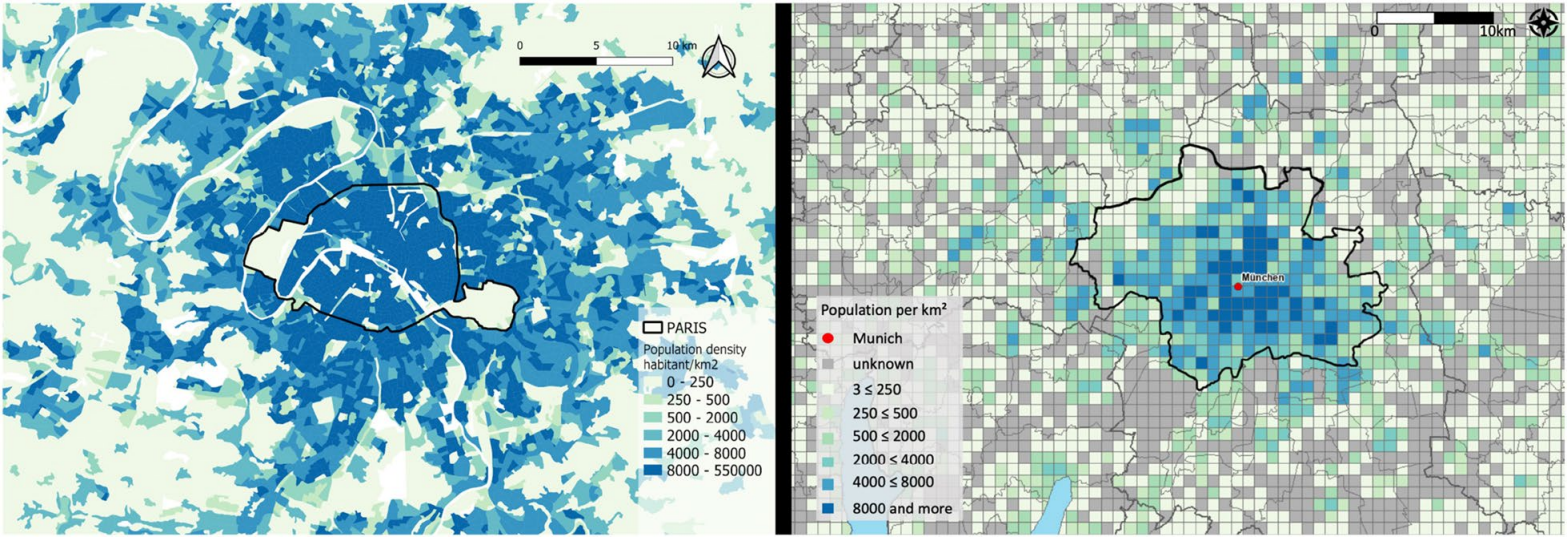
Population density of Paris (left) **a)** and Munich (right)** b)** and the corresponding surrounding area [41, 42]

The two cities differ significantly (Table 1). Paris, the French capital, is the most populous city in France. Conversely, Munich is Germany’s third most populated city, with about 1,5 million inhabitants. On a surface of 310 $\textrm{km}^2$, including some agricultural land, Munich’s population density, meaning the number of people per square kilometer ($\textrm{km}^2$), is relatively low 4.839 inhabitants/$\textrm{km}^2$, compared to Paris with 20.952 inhabitants/$\textrm{km}^2$. By contrast, the European population density was, on average, 109 persons per $\textrm{km}^2$ in 2019 [38]. Urbanization continues within Munich’s city limits, with almost 120 $\textrm{km}^2$ of potential building land, representing 38% of Munich’s territory, as 18% are protected green areas and about 44% are considered sealed surfaces. On the contrary, Paris is the most densely populated city in Europe, with 2,2 million inhabitants living on a surface of only 105 $\textrm{km}^2$, leaving no more space to expand but beyond the city limits. The City of Paris only encompasses the inner core of a very densely populated metropolitan area (Fig. 1a). The Parisian metropolitan area extends far beyond Paris’ administrative boundaries, with more than 12 million inhabitants living in the total Île-de-France area [39]. More than 1 million employed people commute daily to the city center for work [40]. They are responsible for additional fossil fuel carbon emissions that are partly not accounted for when only direct emissions within the administrative boundaries are considered. However, since the Paris Climate Plan only applies to the city’s inner core (City of Paris, black line in Fig. 1a), we focus in this study on the same area for consistency.

Paris and Munich have implemented two fundamentally different strategies to reach climate neutrality. The most striking contrast arises from their geographical constraints: as mentioned above, the city of Paris has nearly reached its maximum density, as no land is left for new constructions (except urban parks), and the building height remains restricted [43]. To achieve climate neutrality, the city of Paris targets renovating existing residential and commercial buildings, and clean mobility, for which for example, it foresees a ban on all thermal-powered cars by 2030. Also, Paris strives for a completely renewable (and recovered) energy consumption, out of which 20% should be produced locally by 2050 (including waste incineration, solar, wind, and biomass). Conversely, Munich still has space to expand in the future (Fig. 1b) and already has plans for future residential areas. Germany’s, and consequently also Munich’s energy mix, with coal and natural gas representing about 45% of the total energy mix, is fundamentally different from the nuclear, low-emissions energy mix in France (nuclear representing about 75% of the total primary energy production) [44]. Indeed, when having a closer look at the carbon intensity of both countries, the difference is striking. The energy consumption in France, taking into account energy imports and exports, accounts for a carbon intensity of 90 g$\textrm{CO}_2$eq/kWh over the year 2022, while Germany reports a carbon intensity of 473 g$\textrm{CO}_2$eq/kWh in 2022 [45]. France shows a 3,6 times lower carbon intensity of electricity produced and used than the EU27 member states together in 2019, while Germany has a 30% higher carbon intensity than the EU27 [46]. Therefore, Munich and its 100% owned energy company SWM mainly focus on power transformation, particularly on phasing out fossil fuels to renewable energies, while using gas as a transitional solution. This change comprehends the shift from coal to gas at Unit 2 of the North Power Plant in Unterföhring (Unit 2 in 2018: 1346.2kt$\textrm{CO}_2$) [47] and the development of a geothermal heating and cooling system that uses underground streams [48]. Munich also puts its efforts on the tertiary sector, which has always been the most emitting sector according to the city inventory [49], and on traffic, for which it envisages an increase in bicycle use.

## Methods

This study aims to map future fossil fuel emissions to provide essential information for atmospheric monitoring systems. The study was conducted over two European cities of different sizes, Paris (France) and Munich (Germany). The Netherlands Organisation for Applied Research (TNO) provided the spatially distributed 1 km x 1 km $\textrm{CO}_2$ inventory for 2019, on which future emissions will be computed. This computation of future emissions is based on the analysis of the corresponding Climate Action Plan of each city.

### TNO Spatially-resolved emission inventory

GHG emissions inventories remain an important source of information that allows governments, policymakers, and corporations to i) understand their carbon footprints and ii) develop Climate Action Plans in order to reduce their GHG emissions. In this study, we use the 1 km x 1 km resolution activity-based emissions inventory developed by TNO. The TNO inventory has been downscaled from national data to the urban scale using spatial proxies [50]. It is based on nationally-reported emissions to UNFCCC and the European Monitoring and Evaluation Programme (EMEP), spatially distributed according to various proxies such as population density and road network maps. The  1 km x 1 km version was prepared within the European CHE and VERIFY projects (https://verify.lsce.ipsl.fr/). The inventory only considers Scope 1 emissions, meaning only emissions caused by burning fossil fuels and biofuels on the city’s territory. Hence, emissions caused by energy transformation are attributed to their respective energy facilities rather than the energy consumption locations.

### Comparison of TNO to the official cities’ inventories

To evaluate the emissions from our spatially-resolved inventory, we compared the city SRIs to the TNO emissions estimates over the same area (city boundaries). We present details of the reconciliation in the Supp. Material (see Additional File 1). For Munich, the two inventories do not display the same total for the year 2019, due to different sector definitions and methodologies and due to the different greenhouse gases ($\textrm{CO}_2$ and $\textrm{CO}_2$e) they considered. TNO estimates 7.256.910 tons $\textrm{CO}_2$ and Munich 7.956.214 tons $\textrm{CO}_2$e (including $\textrm{CO}_2$, CO, $\textrm{CH}_4$, and $\textrm{N}_2$O). Moreover, the definition of the sectors differs widely from another (see Additional File 1). First, the tertiary sector within Munich’s inventory includes emissions from small businesses with up to 19 employees [51]. Small companies with less than ten employees represent a share of 90% of the total number of companies based in Munich. Large industries account for just 10% of the city’s corporate emissions [52]. Second, for the traffic sector, the two inventories differ essentially. The city of Munich calculates its traffic emissions based on the national TREMOD (Transport Emission Model) traffic model, which accounts for the traffic volume, differentiated by vehicle types, street types (hence the speed of vehicles), and fuel types, within the territorial limits. TNO traffic emissions are based on the national total and downscaled based on proxy data, such as road properties, traffic volume, and fleet composition. In a comparison study, it is mentioned that the TNO inventory might need adjustment in the spatial distribution of its traffic emissions as a systematic underestimation of emissions from road transport in urban areas has been noticed in Paris and Zurich (pers. comm.). Third, Munich’s inventory considers the municipality as a sector, whereas this sector is not singled-out in the TNO categorization but is rather included in the TNO stationary combustion category. Lastly, the TNO inventory provides information on the public power sector, which the Munich inventory distributes in each corresponding sector. For Paris, the two inventories do not display the same total either. For the year 2018 (last year Paris published its inventory) the TNO inventory indicates about 30% less total emissions than the Paris SRI. The difference can be attributed to several factors, such as the use of different greenhouse gases ($\textrm{CO}_2$ and $\textrm{CO}_2$e) but also due to different sector definitions and methodologies applied. The city of Paris calculates its greenhouse gas emissions based on the methodology prepared by the French Bilan Carbone^®^Association, estimating the volume of an activity multiplied by a corresponding emission factor, as opposed to the down-scaled inventory by TNO. Greenhouse gas emissions are expressed in $\textrm{CO}_2$ equivalent by the city, whereas TNO provides $\textrm{CO}_2$ only. Besides, the city also reports its emissions on the Carbon Disclosure Project Plateforme in the GPC format, making an inter-city comparison easier. The city differentiates between direct emissions and indirect emissions. Direct emissions include all local emissions from Paris, such as energy consumption in buildings, inner-city transport, and waste. Indirect emissions also consider all additional emissions generated outside of the territory through, for example, food consumption or transportation outside the city boundaries (including flights).

### Projections of gridded $\textrm{CO}_2$ emissions for 2030 and 2050

For the projection of the gridded $\textrm{CO}_2$ emissions for the target years 2030 and 2050, the first step was to reconcile the sectors of the TNO 2019 inventory with the city’s climate action plan. We chose to base the study on the 2019 TNO inventory to avoid the impact of the COVID-19 pandemic on the activity data for the year 2020 and after. The second step was to detail and quantify the reduction targets based on the climate plans, and the third was to subtract those avoided emissions from each sector in order to create future emission maps.

**Fig. 2:**
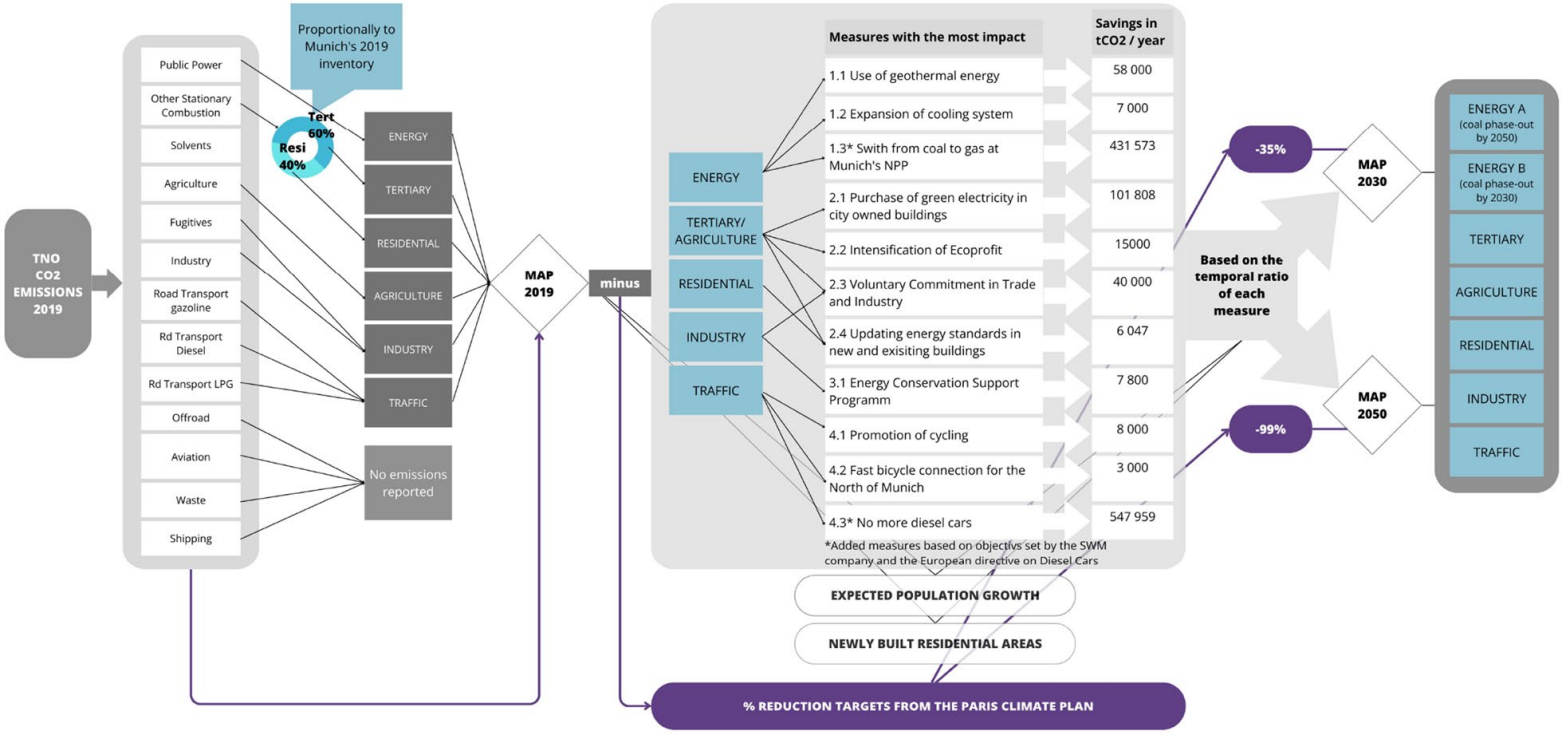
Flowchart of the applied methodology

For Munich, sector harmonization was especially important as the TNO sector classification differs from the Munich sectors. Therefore, Munich’s climate action items were re-distributed according to the TNO inventory sectors. TNO uses a GNFR sector classification [50, 53], whereas the city of Munich’s inventory divides emissions among five sectors (Industry, Tertiary, Residential, Traffic, and Municipality). For the harmonization, we applied the following changes:

- Other Stationary Combustion was split proportionally among the residential and tertiary sectors, based on their respective weights in the city inventory of 2019 [49]
- Solvents are added to Residential, based on the Emission inventory guidebook [54]
- Fugitives emissions are added to Industry, on the same basis as for Solvents [55]

Next, we analyzed Munich’s Climate Plan (IHKM- “Integriertes Handlungsprogramm Klimaschutz München“) [56]. The IHKM of 2019 contains 113 measures, of which eight have been disregarded since these measures target emissions outside the city’s boundaries. An exception was made for measure 1.3 (Fig. 2), the switch from coal to natural gas at Unit 2 of the North Power plant, since the powerplant is only 700 meters outside the city limits and represents the largest single source of $\textrm{CO}_2$ emissions in the city. Therefore, it has been included in the study. We also added measure 4.3 (Fig. 2), based on the recent debate within the EU [57] and Munich’s mobility strategy [58]. Measure 4.3 forbids the sale of diesel cars starting in 2035. We hypothesize that measure 4.3 will be implemented, at the latest, by 2050. The corresponding $\textrm{CO}_2$ emission reductions are quantified based on TNO’s energy types within the traffic sector (gasoline, LPG, and diesel). Only half of the measures in the IHKM have been quantified in terms of annual $\textrm{CO}_2$ savings (cf. Results). We define the implementation timeline as indicated in the Climate Action Plan for each quantified mitigation measure, including the realization period and the duration of the specific action. We added annual $\textrm{CO}_2$ savings for each year over the effective periods, starting after its implementation, and then presented the accumulated impact for the years 2030 and 2050. In the flowchart (Fig. 2), we listed the most impacting measures, grouped by sector, representing 98% of the total quantified reduction measures. Two additional factors have been included in the computation of future emissions: population growth and new residential areas. The latter comprises three future residential areas: Neufreimann (planned for 2030), Freiham (planned for 2030), and Nordosten (planned for 2050) [59]. Once the areas of these future residential zones were located, we updated the local per capita emissions according to the estimated future population at the city scale. This methodology allowed us to project the total emissions for 2030 and 2050. We also divided our projections into two scenarios (Scenario A and Scenario B), two plausible scenarios for the realization period of measure 1.3 (North Power Plant coal-to-NG switch). Munich aims to shut down the coal unit before winter of 2023/2024, which is still in operation. Initially, the shutdown had been foreseen for the winter season of 2022. Nevertheless, the city council has decided to postpone the shutdown due to the energy crisis following the war in Ukraine [60]. Since the latter increases the uncertainty related to the supply of natural gas and, thus, the dependence on coal. Scenario A foresees the completion of the measure by 2050, whereas scenario B expects this measure to be completed by 2030.﻿

For Paris, a more simplistic approach has been taken, as the quantification of mitigation actions was not available. The current Climate Action Plan (updated in 2018) comprises a list of climate actions across different sectors (e.g., building renovation, construction of a solar power plant, restricted traffic area, etc.), some of them voted at the city council. However, these actions were not quantified. The Paris Climate Plan provides overall relative reduction targets for the years 2030 and 2050. Therefore, the methodology applied to map future emissions over Paris uses the total relative reduction targets applied to the sectoral emissions from the 2019 TNO inventory, as shown in the flowchart (Fig. 2). Similar to Munich, the 2019 TNO inventory is used as a baseline to compute the future emissions of Paris. The City of Paris aims to achieve a relative reduction of 50% of its direct emissions by 2030 compared to 2004 [61], leaving Paris with a reduction target of 35% from 2019 to 2030 (Fig. 5b). By 2050, Paris aims to achieve climate neutrality by decreasing its total (indirect + direct) emissions by 80% and offsetting the remaining carbon emissions. Regarding direct emissions, the 2050 objective in our study relies on near-zero carbon emissions, converted here into a 99% reduction.

## Results

**Fig. 3:**
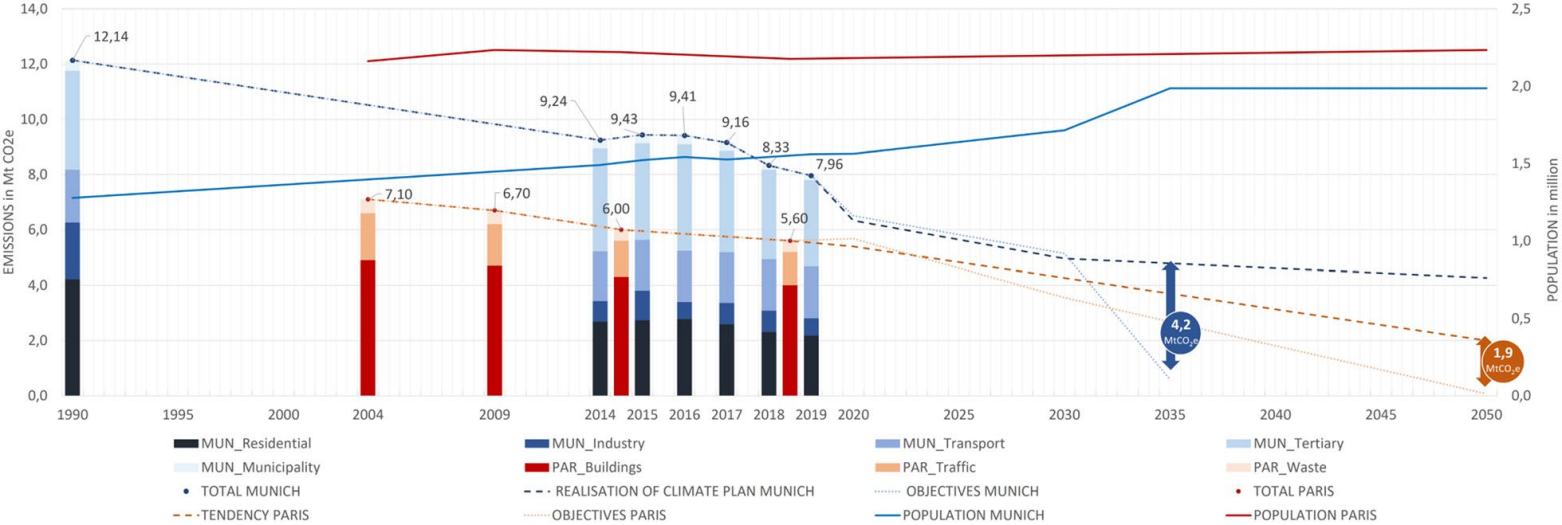
Emission trajectory of the two cities and emission targets

The two cities have very ambitious climate policies to tackle climate change within their administrative boundaries. The past and future emissions for the cities of Paris and Munich are presented in Fig. 3, including the city’s population numbers (solid lines). Munich selected 1990 as its baseline year, whereas the City of Paris selected 2004 as reference. Paris and Munich show a very similar rate of annual emission reduction, with 1,5% per year (between 2004 and 2018) and 1,6% per year (between 1990 and 2019), respectively. The methodology used to establish the original 1990 inventory of Munich could not be precisely identified (standards have evolved rapidly in the last 20 years) but the Paris baseline year (2004) coincides with the year of their first published inventory. It can also be noted that the two cities update their SRIs on different frequencies. The City of Paris publishes an update every 4 to 5 years starting in 2004, whereas Munich started its series of annual updates in 2014. This long-term versus short-term approach is also reflected in their climate plans. Munich’s Climate Plan targets the coming three years, while Paris targets the entire period ending in 2050. Their climate targets differ accordingly: Paris’ objectives are expressed as relative reduction targets compared to the total emissions for the baseline year 2004, while Munich (i) states its overall objectives as per capita emissions and (ii) provides quantified action measures. The stacked bar chart in Fig. 3 illustrates the city emissions from the SRIs in t$\textrm{CO}_2$e, cumulating the corresponding sectors. Munich’s data is represented by blue colors, and Paris by red colors. Starting in the baseline year until the most recently published inventory, which is the year 2018 for Paris and 2019 for Munich [49, 62]. The projected emission trajectory (dashed line) for Munich has been based on the city’s climate plan and the therein quantified reduction measures (as described in the methodology section). For Paris, the past year’s emission reduction trend has been extrapolated until 2050 since no quantification of the mitigation measures is available. The cities’ target trajectories (pointed lines) differ widely with respect to the years to achieve climate neutrality. Munich strives for climate neutrality by 2035, while Paris strives for that target by mid-century. For Munich, the objectives are 3,0 tons $\textrm{CO}_2$e per capita in 2030 and 0,3 tons $\textrm{CO}_2$e per capita in 2035 [63]. For Paris, the objectives are a 50% reduction by 2030 and a 100% reduction by 2050 of local emissions compared to 2004. The population growth reveals a progressive increase for Munich compared to a stabilized average for Paris [64], resulting from urbanization dynamics within the city boundaries (saturated in Paris but not in Munich). When reconciling the two objectives on the same metric, Paris’ target to cut local emissions by 100% by 2050 results in a much lower value (0,03 tons $\textrm{CO}_2$e per capita) than Munich’s target of 0.3 tons $\textrm{CO}_2$e per capita by 2035 (also see Additional file 1). Munich targets to decrease its emissions by a factor of two by 2030 and to reach zero net emissions by 2035, requiring a rapid decline in emissions between 2030 and 2035 (Fig. 3). The quantified mitigation measures for Munich lead us to estimate residual emissions of 2,41 t$\textrm{CO}_2$e per capita by 2035, showing a large gap to the target (Fig. 3). However, the quantification of mitigation actions in Munich is limited to about half of the total actions outlined in the city’s Climate Action Plan, indicating that the city’s actual mitigation potential might be higher than what is presented in this study. Nevertheless, even when doubling the quantified mitigation actions, Munich would still exceed its emissions target and emit 0,85 t$\textrm{CO}_2$e per capita in 2050 (see Additional file 1). In view of the very ambitious goal of climate neutrality for the city as a whole by 2035, it is questionable if this target is reachable since certain long-term infrastructural changes (buildings, transportation system, and energy supply) need time for implementation and cannot be completed at any speed, even in an encouraging environment [65]. For Paris, the absence of quantified climate actions limits our ability to assess the planned trajectories based on the climate plan. However, when extrapolating the past year’s declining trend in emissions to 2050, Paris achieves a reduction of 72% (0,9 t$\textrm{CO}_2$e per capita) compared to the objective of a 100% reduction.

**Fig. 4:**
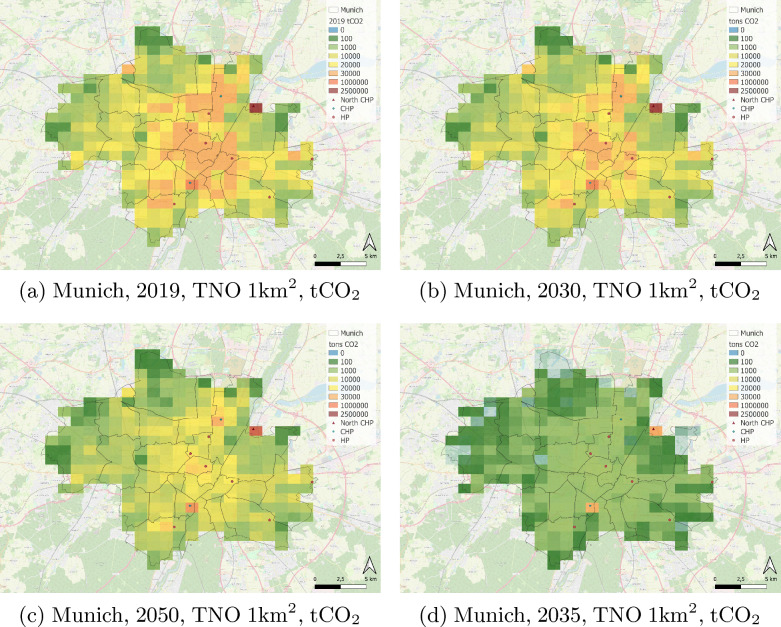
Spatial distribution of the total annual emissions over the city of Munich based on the TNO 1x1km inventory according to the realization of the climate actions (Panel **a**, **b**, **c**). Panel **d** shows Munich in 2035 if the goal of 0.3 tons $\textrm{CO}_2$e per capita is achieved.* CHP* Combined heating and power plant, *HP *Heating plant

As described in the methodology section, we combined the spatialized 2019 TNO 1 km x 1 km inventory with the sectorized mitigation actions from the climate plan to generate projected emissions maps for 2030, 2035, and 2050. The spatial distribution of emissions for Munich is shown in Fig. 4, higher values being associated with high population density areas and the two power plants (East and South of the city). The switch from coal to gas is visible on the map for the year 2050, when the phasing out will most likely be accomplished (possible by 2030 but uncertain). The mitigation efforts planned by the city government are partially offset by an increase in urban population and new residential areas. The outskirts of Munich have traditionally been dominated by agricultural activities (represented by areas in green around Munich in Fig. 4). Remaining emissions can be seen in the city center by 2050, dominated by the tertiary sector. Figure 4d) shows what Munich’s emissions would look like based on the target of 0,3 tons $\textrm{CO}_2$e per capita that the city aims to achieve by 2035. This target is however only achievable through a complete shift to renewable energies, an objective yet to be added to the next Climate Plan. In terms of atmospheric monitoring, our results suggest that i) the core of the city should be closely monitored since most of the projected reductions will take place there, ii) the outskirts of Munich will require additional sensors to track the potential increase in emissions (since new residential areas will be located there) and the envisaged decrease in emissions due to carbon storage and capture by vegetation in protected areas (such as nature reserves).

**Fig. 5:**
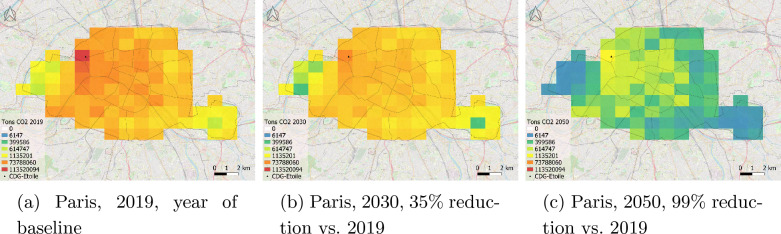
Spatial distribution of the total annual emissions in t$\textrm{CO}_2$ over Paris based on the 2019 TNO 1 km x 1 km inventory **a**) and the climate plan objectives for 2030 **b**), and 2050 **c**)

Regarding the spatial distribution of the Paris emissions, major traffic areas are responsible for the highest emission rates, such as Charles-de-Gaulle/Etoile (black triangle in Fig. 5) in the northwestern part of the city (Fig. 5a). The traffic sector, the largest in the western part of the city, is responsible for the observed gradient in total emissions. The reductions for the year 2030 are, as expected, not as significant as for 2050 (slow carbon storage rates by vegetation and soil) when carbon uptake from urban parks (Vincennes, Boulogne) becomes significant (Fig. 5c). We conclude that Paris, focusing on traffic decrease and building renovation, will likely exhibit a reasonably homogeneous reduction in emissions over its territory despite the lack of quantified action items. We note that the scenario based on the climate objectives of Munich (Fig. 4d) uses the same methodology as for Paris 2050 emissions (Fig. 5c). The lack of quantified climate actions in Paris limits our ability to determine an optimal network density. Future research may be needed to design an optimal observation network. Such a future observation network should also consider stations in urban parks, where there might only be a limited uptake of $\textrm{CO}_2$ observed due to roads and traffic passing through those green areas. In order to design the future observation network and take into account the sources and sinks within the city, accurate and well-defined targets of the system are needed.

## Discussion and Conclusion

We have shown how climate actions, population growth, and urbanization produce mixed spatial patterns across two European cities. In terms of atmospheric monitoring, our results demonstrate the need for additional measurement stations located inside the densest areas of the two cities but also in the outskirts of the urban core (Munich) and within urban parks located near the city boundaries (Paris). Significant changes in emissions will correspond to increases (urbanization of agricultural and forested land) and decreases (traffic control on the beltway).

The quantification of individual climate actions and projection of carbon emissions face significant uncertainty since their application depends greatly on political will, social acceptance, and financial feasibility. Consequently, the projected emissions presented in this study provide only a partial understanding of what future emissions might entail due to evolving circumstances and dynamic socio-political contexts. While Munich has quantified a significant fraction of its planned mitigation measures in the Climate Plan of 2019, the latest version of 2022 only provides sectoral objectives, similar to most European cities. Nonetheless, the fact that there is no official requirement to quantify action items is one of the primary reasons that cities do not make such an attempt. Paris has defined its long-term objectives but does not provide quantified emission reductions for each policy measure. Moreover, these measures only apply to the municipality as defined by its administrative boundaries. Focusing solely, on the one hand, on the administrative boundaries assures comparability and consistency, as well as direct applicability of the Climate Action Plan. On the other hand, this artificially narrows the scope of the study and does not consider the dynamic interactions with the surrounding areas. In Paris, the inner city population represents only a fraction of the total population living in the Paris metropolitan area. For instance, over 1 million employed people commute to Paris for work on a daily basis [40], requiring coherent climate actions in neighboring cities to effectively reduce traffic emissions beyond administrative boundaries. Spatialized information is therefore needed to better target mitigation strategies.

We show that the two cities have pledged ambitious mitigation objectives to reach climate neutrality by 2035 (Munich) or by 2050 (Paris). While the overall goals remain similar (50% reduction by 2030), the lack of quantification impairs our ability to assess the feasibility of the Paris Climate Plan and results in less differentiated spatial patterns for future emissions. Recently, Munich’s city council moved its original climate neutrality target from 2050 to 2035. Because carbon neutrality differs from net zero emissions, the remaining carbon emissions will be offset by carbon sequestration or through carbon credits. For Paris, about 20% of its remaining emissions will be compensated by carbon compensation mechanisms in 2050. A recent study [66] estimated that the city of Paris, projected to emit about 2,5 million tons of $\textrm{CO}_2$e by 2050, would need a total area of about 10.000 $\textrm{km}^2$ to compensate Paris’ emissions with afforestation, equaling the current surface area of the Île-de-France region. Furthermore, the two cities use different metrics to measure their climate targets. While Munich uses per capita emissions, Paris targets relative emission reductions. The climate plans, as well as any measurement of progress and success, are thus incomparable. Other European cities might even have additional metrics to reach their climate targets. A standardized European crosswalk table is needed to disentangle European city strategies, enabling the inter-comparison of the various strategies.

Reality further shows that cities do not have all means in their hands to reduce their emissions as much as they would like to. Administrative boundaries, access to reliable emission data, and the lack of deep knowledge of mitigation and adaptation actions may lead to an incomplete policy assessment. Local governments have limited political power to control emissions within their jurisdictions, as they only have an impact on 10% to 15% of the global share of emissions [67]. When considering Scope 3 emissions, encompassing emissions from food imports, consumption, and building materials, then the influence of local governments through incentives and informational measures could account for up to 50% of the overall impact on emissions reduction. Meanwhile, national governments withhold political influence over local authorities and administrations. Across Paris, mayors are elected in each of the twenty districts (arrondissements), governing the use of cooling and heating systems but with limited control over traffic. In addition, the public transportation system of Paris is managed by the region Île-de-France, limiting the city government’s ability to modify the bus capacity or to build new metro lines. By contrast, the city government of Munich is responsible for the city’s traffic sector, including public transportation, except for the S-Bahn and the regional railway, both under the authority of the Bayern region [68]. It also holds political power over its energy sector, provided that the source of emissions is located within the city boundaries [60]. Therefore, the synergy among different governance levels remains critical [14] to achieve climate targets, particularly when cities are engaged in ambitious climate objectives, often more ambitious than national climate targets.

Our study is limited by the emissions inventory of 1 km x 1 km. Future research will consider projected emissions at finer spatial scales, down to the building-level, to enable a more granular spatial analysis and provide more localized information on emissions trends, especially per sector for local decision-makers. This first study demonstrated the complexity of intra-urban emissions changes, but future structural changes should be introduced based on detailed urban planning. Future atmospheric networks should be built on the spatialized information provided by the projected emissions maps. The city of Paris has engaged in replacing current highways with underground highways, which can significantly impact the spatial distribution of emissions. Similarly, the City of Paris pursues gradually-increasing restrictions on diesel and gasoline cars in the city, likely to shift car commuters to other transportation methods (public transportation, bikes, electric cars) [61]. Future research is also needed to better understand the increasing influence of heat waves, leading to a possible increase in air conditioning deployment, as already observed in Paris [69]. We conclude here that a more quantitative assessment of climate actions and their indirect impacts would help strengthen climate policies and their effectiveness at the city level.

### Supplementary Information

**Additional file 1: Figure S1.** Comparison of the sectoral distribution within the TNO inventory to the city inventory of Munich for 2019 (top panel) and of Paris for the year 2018 (bottom panel).** Figure S2.** Munich's emissions as a relative difference between 2019 and 2050 (*CHP *Combined heat and power plant, *HP *Heat plant). **Figure S3.** GHG emission scenarios for Munich based on the Climate Action items applied to the city inventory (in tCO2e/capita). The actual city targets are indicated in green for 2030 and 2050, in parallel with an enhanced mitigation scenario (mitigation measures x2) in light blue. **Table S1.** GHG emissions per capita split by sector in Munich in kt CO2e. **Table S2.** TNO 2019 sectoral emissions, emission savings from Munich's climate plan, projected emissions for Munich for 2030 and 2050. **Table S3.** Crosstable of Paris' and Munich's emission targets for 2030 and 2035 (Munich) and 2050 (Paris).

## Data Availability

The data is available either in the manuscript itself or in the listed references. Additional data may be requested from the corresponding author.

## Abbreviations

GHG: Greenhouse gas(es)
IPCC: Intergovernmental panel on climate change
SDG: Sustainable development goals
SWM: Stadtwerke Muenchen
SRI: Self-Reported inventories
TNO: Netherlands organisation for applied scientific research
UNFCCC: United Nations Framework convention on climate change
CLRTAP: Convention on long-range transboundary air pollution
BISKO: Bilanzierungssystematik kommunal
GNFR: Gridded nomenclature for reporting
EEA: European environmental agency
IHKM: Integriertes Handlugsprogramm Klimaschutz Muenchen
