# Supplementary material for: From political pledges to quantitative mapping of climate mitigation plans: Comparison of two European cities
Source: Carbon Balance Manag. 2023 Sep 6;18:18. doi: 10.1186/s13021-023-00236-y (PMC10481584; doi:10.1186/s13021-023-00236-y)
Supplement: Supplementary file 1 — Additional file 1: Figure S1. Comparison of the sectoral distribution within the TNO inventory to the city inventory of Munich for 2019 (top panel) and of Paris for the year 2018 (bottom panel). Figure S2. Munich's emissions as a relative difference between 2019 and 2050 (CHP Combined heat and power plant, HP Heat plant). Figure S3. GHG emission scenarios for Munich based on the Climate Action items applied to the city inventory (in tCO2e/capita). The actual city targets are indicated in green for 2030 and 2050, in parallel with an enhanced mitigation scenario (mitigation measures x2) in light blue. Table S1. GHG emissions per capita split by sector in Munich in kt CO2e. Table S2. TNO 2019 sectoral emissions, emission savings from Munich's climate plan, projected emissions for Munich for 2030 and 2050. Table S3. Crosstable of Paris' and Munich's emission targets for 2030 and 2035 (Munich) and 2050 (Paris). [file 13021_2023_236_MOESM1_ESM.pdf]

Additional file

From political pledges to quantitative mapping of climate mitigation plans: comparison of two European cities

Reconciliation of the Climate Action Plan nomenclature with the inventories

The terminology used for the estimated reductions from individual action items within Munich's climate action plan differs from the TNO inventory nomenclature. While Munich's city inventory follows the German BISKO standard methodology provided by the German Ministry of environmental affairs [1], the TNO inventory aggregates emissions to the GNFR (Gridded Nomenclature for Reporting) level, which lists the following sectors:

1	A	A_PublicPower
2	B	B_Industry
3	C	C_OtherStationaryComb
4	D	D_Fugitives
5	E	E_Solvents
6	F1	F_RoadTransport_exhaust_gasoline
6	F2	F_RoadTransport_exhaust_diesel
6	F3	F_RoadTransport_exhaust_LPG_gas
6	F4	F_RoadTransport_non-exhaust
7	G	G_Shipping
8	H	H_Aviation
9	I	I_OffRoad
10	J	J_Waste
11	K	K_AgriLivestock
12	L	L_AgriOther

For Munich, the latest emissions inventory of the city from August 2022 includes the direct emissions of the main GHGs (CO_2, CH4, N2O …), adjusting each gas species by its relative global warming potential compared to CO_2 (CO_2 equivalent, or CO_2eq). Emissions are divided into five activity sectors: residential, tertiary, traffic, industry, and municipality. The BISKO standard uses a territorial approach, hence referring to emissions occurring within the city's administrative boundaries, excluding, for instance, Munich's international airport. For stationary energy consumption, the sectors recommended by BISKO are industry, tertiary, residential, and municipal facilities. We note that energy consumption on the territory is allocated to each sector and the various energy sources. Within energy sources, the BISKO standard recommends considering both distribution pipelines

and non-pipeline (transmission facilities,...) energy sources. With that methodology, biofuels are considered in conformity with the TNO inventory. To calculate CO_2eq, the BISKO Standard takes into account the national electricity mix. For the traffic sector, the BISKO Standard includes emissions from motorized transport within the municipal boundaries based on traffic volumes occurring within their territory.

The sector comparison of the two inventories is as follows:

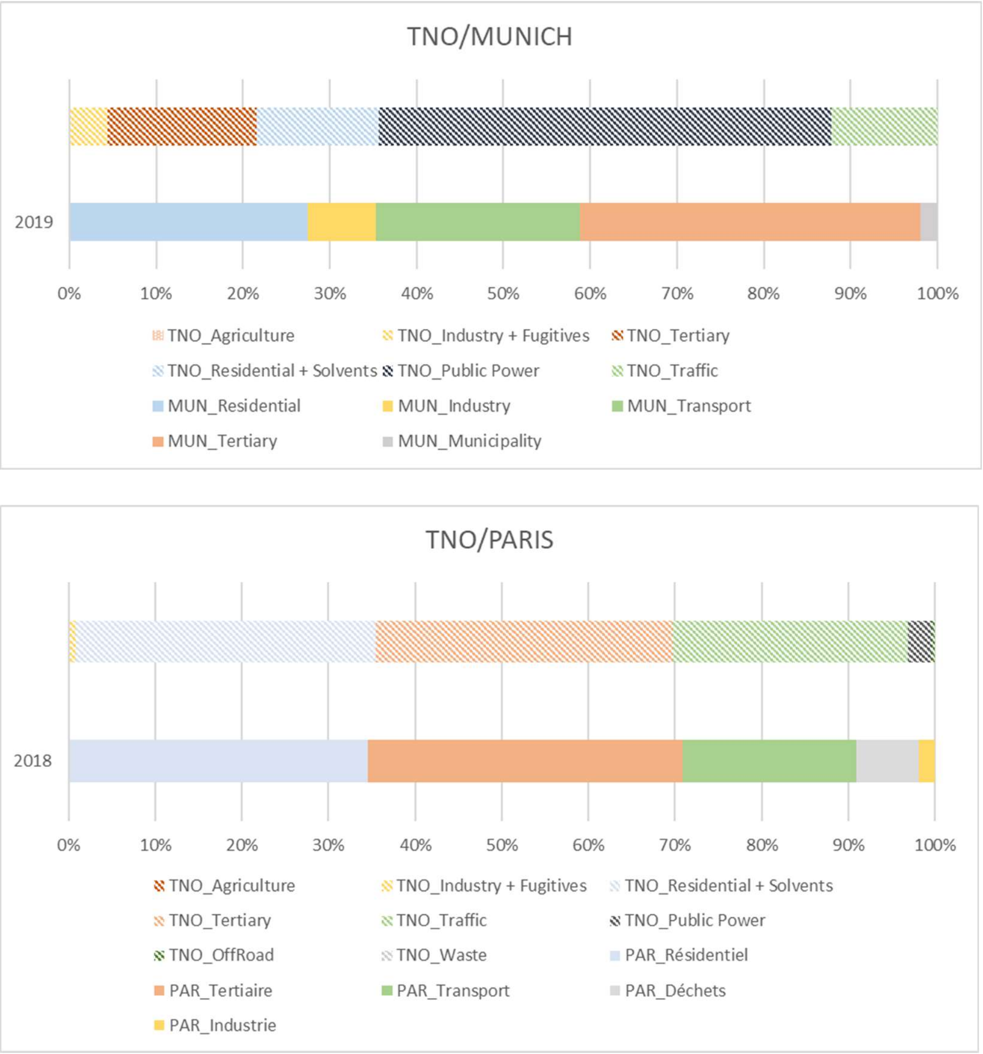

Figure 1: *Comparison of the sectoral distribution within the TNO inventory to the city inventory of Munich for 2019 (top panel) and of Paris for the year 2018 (bottom panel).*

The following assumptions were made to make the two inventories comparable in order to apply the city's mitigation actions to the TNO inventory:

- A_Public_Energy as the Energy Transformation sector of the Munich Climate Plan.
- Sector B_Industry is comparable to Industry in Munich's Climate Plan.
- Sector C_ OtherStationaryComb is composed of both the residential and tertiary sectors. Therefore, we split the C_OtherStationaryComb, based on the relative weight of the residential and tertiary CO_2 emissions in Munich's 2019 inventory. The resulting split of Sector C accounts for 44% of residential and 56% of tertiary.
- We considered sector D_Fugitive in the industrial sector. [2]
- We considered sector E_Solvents in the residential sector. [3]
- Sector F_Road transportation is comparable to the Road Transport sector of Munich's Climate Plan.
- The G_Shipping and H_Aviation sectors did not cause CO_2 emissions in the TNO inventory, respectively, because of the geographical location of the city of Munich and of the airport, which is located outside of the city's boundaries.
- The I_Offroad sector was not considered, as the Climate Plan calls for increased bicycle use in the city. Therefore, no significant impact on Off_Road emissions.
- There are no reported emissions in the TNO J_Waste sector.
- In Munich's inventory, emissions from the agricultural sector were included in the tertiary sector. To separate the sectors, we calculated the percentage weight from K_AgriLivestock and L_AgriOther on the tertiary + agriculture emissions to produce two different maps for these sectors for 2030 and 2050.

SECTORS	1990	2014	2015	2016	2017	2018	2019
Residential	3,3	1,8	1,8	1,8	1,7	1,5	1,4
Industry	1,6	0,5	0,7	0,4	0,5	0,5	0,4
Transport	1,5	1,2	1,2	1,2	1,2	1,2	1,2
Tertiary	2,8	2,5	2,3	2,5	2,4	2,1	2
Municipality	0,3	0,2	0,2	0,2	0,2	0,1	0,1
**TOTAL**	**9,5**	**6,2**	**6,2**	**6,1**	**6**	**5,4**	**5,1**

Table 1: *GHG emissions per capita split by sector in Munich in kt CO2e*

SECTOR_TNO_NAME	TNO EMISSIONS 2019 kg	Savings		CO2 EMISSIONS in kg	
		2020-2030	2030-2050	2030	2050
PublicPowerA	3 748 349 090	91 219 000	1 171 434 314	3 657 130 090	2 485 695 776
PublicPowerB	3 748 349 090	522 792 314	739 861 000	3 225 556 776	2 485 695 776
RoadTransport	876 292 999	141 027 000	639 755 360	735 265 999	95 510 639
Tertiary	1 238 709 889	557 792 880	166 346 400	681 320 830	515 094 858
Residential	973 272 055	103 408 120	226 053 600	873 592 291	655 689 001
Agriother	897 429	403 821	120 429	493 608	373 179
Fugitives	443	58	-	385	385
Solvents	36 403 620	3 728 356	8 150 310	32 675 264	24 524 955
Waste					
Aviation					
Industry	311 784 886	40 800 000		270 984 944	270 984 944
**Total Scenario A**	**7 185 710 410**	**938 379**	**2 211 860**	**6 251 463**	**4 047 874**
**Total Scenario B**	**7 185 710 410**	**1 369 953**	**1 780 287**	**5 819 890**	**4 047 874**

Table 2: *TNO 2019 sectoral emissions, emission savings from Munich's climate plan, projected emissions for Munich for 2030 and 2050*

After the harmonization of the inventories, the mitigation potential of Munich's climate action plan is attributed to the corresponding TNO sectors from the TNO_GHGco_1x1km_2020_v1_0 dataset, generating 1-km resolution maps for the different TNO sectors.

Munich's 2019 climate plan included 113 measures, of which nearly half have been quantified by an external consulting company. For each of the measures that have been quantified, we used the implementation timeline for each action to apply a linear annual fraction of reduction between its starting date and its target year, resulting in the projection of emissions maps for both 2030 and 2050.

	2030		2035/2050	
	% (vs 2004)	tCO_2e/capita	% (vs 2004)	tCO_2e/capita
Paris	**50%**	1,61	**100%**	0,03
Munich	51%	**3**	94%	**0,3**

Table 3: *Crosstable of Paris' and Munich's emission targets for 2030 and 2035 (Munich) and 2050 (Paris)*

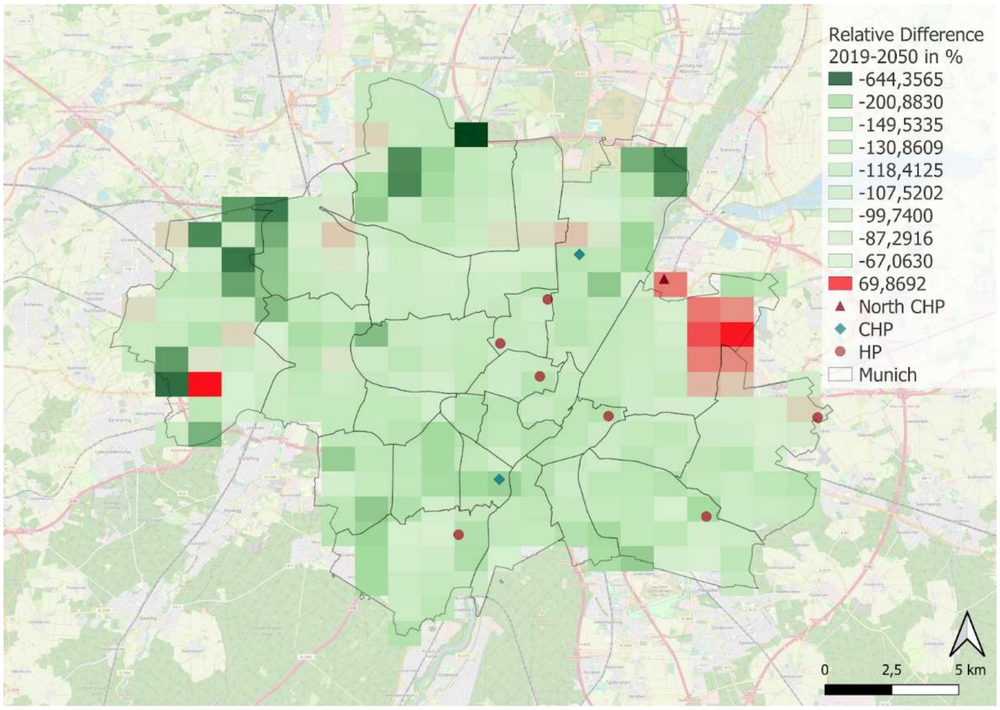

Figure 2: *Munich's emissions as a relative difference between 2019 and 2050 (CHP = Combined Heat and Power Plant; HP = Heat Plant)*

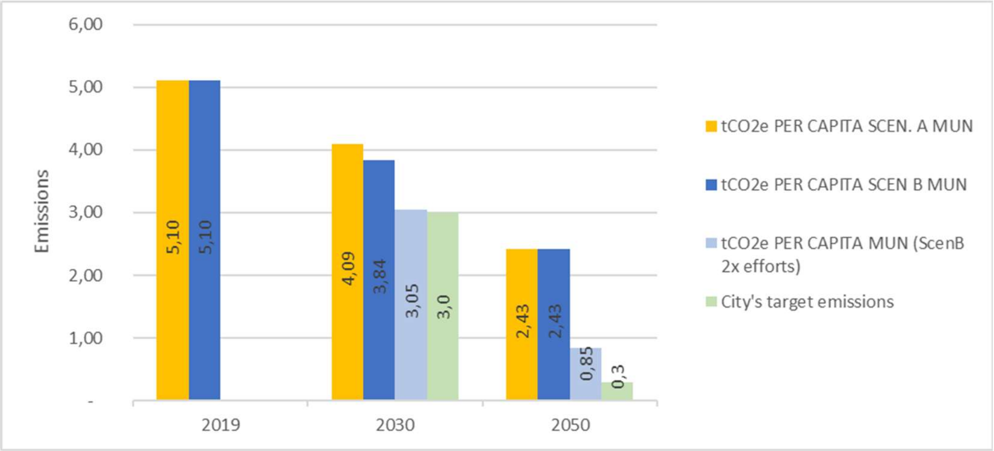

Figure 3: *GHG emission scenarios for Munich based on the Climate Action items applied to the city inventory (in tCO2e/capita). The actual city targets are indicated in green for 2030 and 2050, in parallel with an enhanced mitigation scenario (mitigation measures x2) in light blue.*

2019 in MtCO2	Munich SRI	Munich TNO	Paris SRI	Paris TNO
Industry	0,624	0,312	0,11	0,032
Tertiary	3,120	1,239	2	1,295
Residential	2,184	1,010	1,9	1,306
Traffic	1,872	0,947	1,15	1,027
Waste	0,000	0,000	0,434	0,000
Other	0,156	3,748		0,117
**TOTAL**	**7,956**	**7,256**	**5,594**	**3,778**

Table 4: *Activity-based CO_2 emissions inventory provided by the city (SRI) and TNO in 2019 for Munich and Paris, respectively. (sources: Munich SRI: https://www.muenchen-transparent.de/dokumente/7222928; Paris SRI: https://cdn.paris.fr/paris/2020/02/06/dc2edb10d13ae050815850f721f5a837.pdf)*

REFERENCES

1. Umweltbundesamt: Weiterentwicklung des kommunalen Bilanzierungsstandards f ̈ur THG-Emissionen (2020). https://www.umweltbundesamt.de/sites/default/files/medien/479/publikationen/cc_19-2020_endbericht_sv-gutachten_bisko.pdf, Accessed: 21 Nov 2022
2. EEA: EMEP/CORINAIR Emission Inventory Guidebook - 2007 (2016). https://www.eea.europa.eu/publications/EMEPCORINAIR5/page014.html Accessed 21 Nov 2022
3. EEA: EMEP/CORINAIR Emission Inventory Guidebook - 2007 (2016). https://www.eea.europa.eu/publications/EMEPCORINAIR5/page015.html Accessed 21 Nov 2022
